# Screening for physical inactivity among adults: the value of distance walked in the six-minute walk test. A cross-sectional diagnostic study: Triagem para inatividade física em adultos: o valor da distância percorrida no teste de caminhada de seis minutos. Um estudo transversal diagnóstico

**DOI:** 10.1590/1516-3180.2015.00871609

**Published:** 2014-10-28

**Authors:** Evandro Fornias Sperandio, Rodolfo Leite Arantes, Rodrigo Pereira da Silva, Agatha Caveda Matheus, Vinícius Tonon Lauria, Mayara Silveira Bianchim, Marcello Romiti, Antônio Ricardo de Toledo Gagliardi, Victor Zuniga Dourado

**Affiliations:** I PT, MSc. Doctoral Student in the Department of Human Movement Sciences, Universidade Federal de São Paulo (Unifesp), Santos, São Paulo, Brazil. Universidade Federal de São Paulo Department of Human Movement Sciences Universidade Federal de São Paulo Santos São Paulo Brazil; II MD, PhD. Researcher in the Department of Cardiovascular Medicine, Angiocorpore Institute of Cardiovascular Medicine, Santos, São Paulo, Brazil. Department of Cardiovascular Medicine Angiocorpore Institute of Cardiovascular Medicine Santos São Paulo Brazil; III PE. Master's Student in the Department of Human Movement Sciences, Universidade Federal de São Paulo (Unifesp), Santos, São Paulo, Brazil. Universidade Federal de São Paulo Department of Human Movement Sciences Universidade Federal de São Paulo Santos São Paulo Brazil; IV PT. Associate Professor of the Department of Human Movement Sciences, Universidade Federal de São Paulo (Unifesp), Santos, São Paulo, Brazil. Universidade Federal de São Paulo Department of Human Movement Sciences Universidade Federal de São Paulo Santos São Paulo Brazil

**Keywords:** Motor activity, Physical fitness, Accelerometry, ROC curve, Body mass index.

## Abstract

**CONTEXT AND OBJECTIVES:**

Accelerometry provides objective measurement of physical activity levels, but is unfeasible in clinical practice. Thus, we aimed to identify physical fitness tests capable of predicting physical inactivity among adults.

**DESIGN AND SETTING:**

Diagnostic test study developed at a university laboratory and a diagnostic clinic.

**METHODS:**

188 asymptomatic subjects underwent assessment of physical activity levels through accelerometry, ergospirometry on treadmill, body composition from bioelectrical impedance, isokinetic muscle function, postural balance on a force platform and six-minute walk test. We conducted descriptive analysis and multiple logistic regression including age, sex, oxygen uptake, body fat, center of pressure, quadriceps peak torque, distance covered in six-minute walk test and steps/day in the model, as predictors of physical inactivity. We also determined sensitivity (S), specificity (Sp) and area under the curve of the main predictors by means of receiver operating characteristic curves.

**RESULTS:**

The prevalence of physical inactivity was 14%. The mean number of steps/day (≤ 5357) was the best predictor of physical inactivity (S = 99%; Sp = 82%). The best physical fitness test was a distance in the six-minute walk test and ≤ 96% of predicted values (S = 70%; Sp = 80%). Body fat > 25% was also significant (S = 83%; Sp = 51%). After logistic regression, steps/day and distance in the six-minute walk test remained predictors of physical inactivity.

**CONCLUSION:**

The six-minute walk test should be included in epidemiological studies as a simple and cheap tool for screening for physical inactivity.

## RESUMO

**CONTEXTO E OBJETIVOS:**

A acelerometria fornece medida objetiva do nível de atividade física, porém não é viável na prática clínica. Assim, foram investigados testes de aptidão física capazes de predizer inatividade física em adultos.

**DESENHO E LOCAL:**

Estudo de teste diagnóstico, desenvolvido em laboratório universitário e uma clínica de diagnósticos.

**MÉTODOS:**

188 participantes assintomáticos tiveram o nível de atividade física avaliado por acelerometria, ergoespirometria em esteira, composição corporal por bioimpedância, função muscular isocinética, equilíbrio postural em plataforma de força e teste de caminhada de seis minutos. Foram realizadas análise descritiva e regressão logística múltipla, incluindo idade, sexo, consumo de oxigênio, gordura corporal, centro de pressão, pico de torque de quadríceps, distância percorrida no teste de caminhada de seis minutos e passos/dia no modelo como preditores da inatividade física. Adicionalmente, foram determinadas a sensibilidade (S), especificidade (Sp) e área abaixo da curva dos principais preditores por meio de curvas de característica de operação do receptor.

**RESULTADOS:**

A prevalência da inatividade física foi 14%. O número médio de passos/dia (≤ 5357) foi o melhor preditor da inatividade física (S = 99%, Sp = 82%). O melhor teste de aptidão física foi a distância no teste de caminhada de seis minutos e ≤ 96% dos valores preditos (S = 70%; Sp = 80%). A gordura corporal > 25% também foi significativa (S = 83%, Sp = 51%). Após regressão logística, passos/dia e a distância no teste de caminhada de seis minutos permaneceram preditores da inatividade física.

**CONCLUSÃO:**

O teste de caminhada de seis minutos deve ser incluído em estudos epidemiológicos como ferramenta simples e barata para triagem da inatividade física.

## INTRODUCTION

Physical inactivity is an important risk factor for many diseases, particularly cardiovascular diseases.^1^ It has been suggested that the appropriate level of physical activity is associated with a significant reduction in mortality from all causes.^2^ With aging, the prevalence of physical inactivity increases, thus making its epidemiological evaluation fundamental in designing preventive strategies.^3^

Insufficiently active or totally inactive individuals are those who perform physical activities, but in quantities and at intensities that are insufficient to allow them to be classified as active, since they do not comply with the recommendations of at least 150 minutes/week of moderate to vigorous physical activity.^4^

Questionnaires and self-reporting have been often used to assess the level of physical activity in population-based cohort studies. However, validation studies using accelerometry indicate that the accuracy of the questionnaires is limited, especially in estimating physical activity of milder intensity. Thus, questionnaires may also result in information recall bias.^5^

Alternatively, motion sensors are instruments that are used to detect body movement and can be used to objectively quantify the level of physical activity for a period of time. However, assessment of physical activity within daily life by means of motion sensors is not feasible in clinical practice because the equipment is expensive and the evaluation takes several days to be completed. Another widespread concern around motion sensors is adherence to the evaluation, although this only requires simple care from the individual.^6^

Walking tests have been shown to be closely related to activities of daily life and has been applied to older individuals with and without chronic diseases because of their simplicity with less cognitive demand. Whether functional exercise capacity assessed by field walking tests might be useful for predicting physical inactivity requires further clarification, especially among healthy participants in the general population. Moreover, it has not yet been determined which physical fitness test for screening for physical inactivity would be the most suitable.

## OBJECTIVE

We aimed to identify the best physical fitness test capable of predicting physical inactivity in adults.

## METHODS

One hundred and eighty-eight participants (mean age: 41 ± 14 years; 91 men) used an accelerometer (Actigraph GT3x+) for seven days. The participants were selected from the EPIMOV study (Epidemiological Study of Human Movement and Hypokinetic Diseases). Briefly, the EPIMOV study is a population-based cohort study with the main objective of investigating the longitudinal association shown by sedentary behavior and physical inactivity in relation to occurrences of hypokinetic diseases, especially cardiorespiratory diseases. The volunteers were selected through dissemination in social networks, folders displayed in the universities of the region, local magazines and newspapers. All participants in the EPIMOV study were potentially eligible to form part of the convenience sample of the present study. In the early clinical evaluation, personal and demographic data were collected and participants with previous self-reported diagnoses of heart disease, lung disease or musculoskeletal disorders were not excluded from the present study.

Physical inactivity was defined as less than 150 min/week of moderate to vigorous physical activity in daily life. We excluded swimmers from the analysis because they did not use the device during their training. We evaluated the cardiopulmonary exercise test, body composition (bioelectrical impedance), isokinetic muscle function of the upper and lower limbs, handgrip strength, postural balance (force platform) and six-minute walk test.

The participants were informed about the possible risks and discomforts of this study and signed a consent form. The local Ethics Committee for Research on Humans approved this study.

### Initial clinical evaluation

In the early clinical evaluation, personal and demographic data were collected. In addition, participants answered the physical activity readiness questionnaire (PAR-Q).^7^ Cardiovascular risk stratification for events during exercise was then performed in accordance with the system of the American College of Sports Medicine (ACSM)^8^ and a respiratory questionnaire based on the American Thoracic Society (ATS) questionnaire was administered.^9^

### Anthropometric and body composition evaluation

Body weight and height were measured and the body mass index (BMI) was calculated. Body composition was determined by means of bioelectrical impedance (310e Biodynamics, Detroit, USA), following the procedure described by Kyle et al.^10^ ^11^ Lean body mass and body fat mass were calculated using the regression equations developed for healthy individuals.^12^

### Cardiorespiratory fitness

Functional exercise capacity was assessed by means of the six-minute walk test, which was performed rigorously in accordance with the American Thoracic Society guidelines.^13^ The six-minute walking distance was recorded in meters and as a percentage of predicted values.^14^

The maximum and symptom-limited exercise capacities were assessed through a cardiopulmonary exercise test (CPET), using a treadmill ramp protocol (ATL, Inbrasport, Curitiba, Brazil). After 3 min at rest, the speed and inclination were automatically incremented in accordance with the estimated maximal oxygen consumption (V'O_2_max), with the aim of completing the test within about 10 minutes.^15^ Cardiovascular, ventilatory and metabolic variables were analyzed breath by breath, using a gas analyzer (Quark PFT, Cosmed, Pavona di Albano, Italy). Oxygen uptake (V'O_2_), carbon dioxide production (V'CO_2_), R (V'CO_2_/V'O_2_), minute ventilation (V'E) and heart rate (HR) were monitored throughout the test. The data were filtered every 15 seconds for further analysis. The anaerobic threshold was obtained in accordance with the standardized v-slope technique.^16^ Two experienced observers independently obtained this index. In cases of disagreement between evaluators, the opinion of a third experienced assessor was considered.

### Balance evaluation

Balance was evaluated from kinetic data at the center of pressure, using a force platform (400 BIOMEC, EMGSystem, Brazil). The frequency of data acquisition on the platform was 100 Hz. The participants were instructed to remain as static as possible, standing with weight borne on both feet, with both eyes open, and then again with both eyes closed. Each position was maintained for 30 seconds.

### Muscle function

Muscle function was assessed using an isokinetic dynamometer (Biodex, Lumex Inc., Ronkonkoma, NY, USA). Peak torque in Nm was evaluated through two trials of five movements at 60°/s. After a rest period of at least three minutes, the participants performed an isometric force test twice, recorded in Nm against fixed resistance over a 60° range of flexion. After another similar rest period, the participants performed 30 movements at 300°/s to record the total work, in kJ. The highest value was selected for analysis in all the abovementioned tests. These tests were applied to the quadriceps femoris and biceps brachii. 

Muscle function was also assessed by means of handgrip strength. The handgrip strength of the dominant hand was assessed using a hydraulic dynamometer (JAMAR), in accordance with the methods described by Mathiowetz et al.^17^ Three measurements were made, with a minimum interval of 30 seconds between them, and the highest value obtained was subjected to analysis.

### Level of physical activity in daily life

The level of physical activity in daily life (LPADL) was assessed using a triaxial accelerometer that had previously been validated (ActiGraph, MTI, Pensacola, FL, USA).^18^ ^19^ ^20^ The participants were asked to wear the device over their dominant hip on an elasticized belt for 7 days. Days of use were considered to be valid if the participants had worn the device for at least 12 h. They were instructed to remove it for water-related activities, such as bathing or swimming, and to remove it at bedtime. The triaxial ActiGraph measures the duration and intensity of physical activity. Only the data from participants who used the accelerometer for at least four valid days were analyzed.

Physical activity in sedentary, low-intensity, moderate-intensity, vigorous and very vigorous strata was defined as described by Freedson et al.^21^ The minimum level of physical activity in terms of quantity and intensity was considered to be 150 min/week of moderate to vigorous physical activity during the monitoring.^15^ ^22^ Individuals who did not reach this level of physical activity were considered to be physically inactive. For descriptive purposes, we also stratified the participants into three categories of amounts of physical activity, as recommended by ACSM,^22^ i.e. less than 30 min/day, 30-59 min/day and 60 min/day or more.

### Statistical analysis

The sample size was calculated using the OpenEpi free tool (openepi.com). Based on our initial experiences from the EPIMOV study, we found that the prevalence of physical inactivity was about 14%. We took this to be the prevalence among the 450,000 residents of the city of Santos, São Paulo, Brazil, where the present study was developed. Assuming a 95% confidence interval for precision, alpha of 0.05 and beta of 0.20, we concluded that 185 participants would be enough to develop the receiver operating characteristic (ROC) curves proposed in the present study.

We firstly conducted a descriptive analysis on the data, which included determination of frequencies, histograms, central trend measurements and variability. In order to identify the best physical fitness index capable of predicting physical inactivity, ROC curves were determined and the area under the curve was calculated as representing good combinations of sensitivity and specificity. Areas under the curve greater than or equal to 0.8 were considered to be excellent values.

We calculated the sensitivity, specificity, positive and negative predictive values and accuracy for each predictor. Sensitivity identifies the proportion of individuals who truly do have the disease (in the case of this study, physical inactivity) and present a positive test result and specificity identifies the proportion of individuals who truly do not have the disease and present a correct negative test result. The positive and negative predictive values, respectively, are the proportions of positive and negative results in statistics and diagnostic tests that are true positive and true negative results.

Reduced models were used as a modeling strategy for logistic regression, using physical inactivity as the outcome variable. The physical fitness variables were included in the model as predictors. The model was also adjusted according to demographic and anthropometric variables and also confounding comorbidities. Odds ratios and the 95% confidence interval of the odds ratios were calculated. The probability of alpha error was set at 5%.

## RESULTS

**Figure 1:**
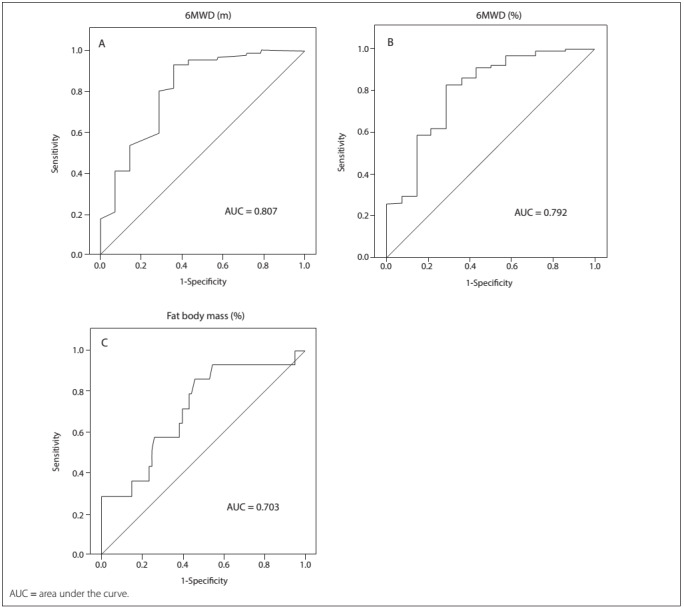
Receiver-operator characteristic (ROC) curves for six-minute walking distance (6MWD) and body fat mass as predictors of physical inactivity.

One hundred and eighty-eight adults aged over 20 years participated in the study (Table 1), and these represented the totality of subjects invited (there were no refusals). Twenty-two percent of the participants performed less than 30 min/day of moderate to vigorous physical activity, whereas 49% performed 30 to 59 min/day and 29% performed 60 min/day or more. As expected, the average number of steps/day (≤ 5357) was the best predictor of physical inactivity (Table 2). The best physical fitness test for predicting physical inactivity was a six-minute walking distance ≤ 511 m (Figure 1A; Table 2) and ≤ 96% of predicted values (Figure 1B; Table 2). Body fat mass > 25% was also significant (Figure 1C; Table 2). All these tests showed high values for the area under the curve. Using multiple logistic regression, the average number of steps/day and the six-minute walking distance remained significant predictors of physical inactivity (Table 3). The correlation between steps/day and six-minute walking distance was moderate but significant (r = 0.415; P < 0.05).

**Table 1:**
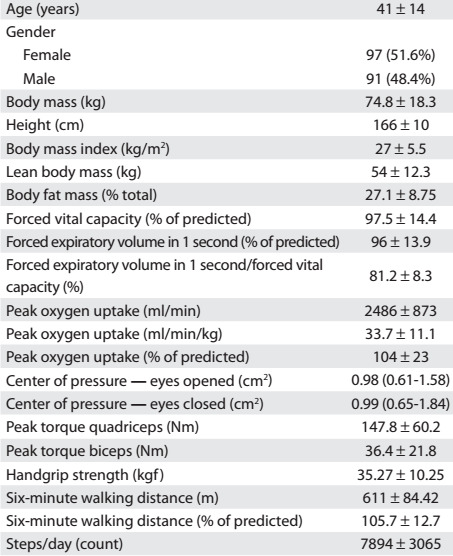
Demographic, anthropometric, lung function, oxygen uptake, static balance, muscle function, walking capacity and physical activity level characteristics of the subjects

Data presented as mean ± standard deviation or as median (with interquartile range).

**Table 2:**
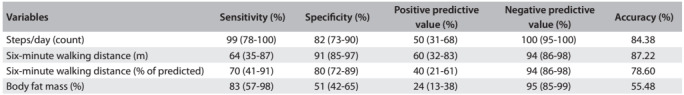
Sensitivity, specificity, positive and negative predictive values and accuracy

Data presented as mean and 95% confidence interval (lower limit - upper limit).

**Table 3:**
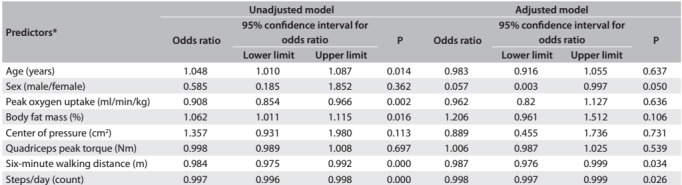
Predictors of physical inactivity after multiple regression analysis

*Model adjusted for race, hypertension, diabetes, dyslipidemia, obesity and smoking.

## DISCUSSION

The present study showed that the six-minute walk test has adequate sensitivity and specificity for diagnosing physical inactivity among adults who are free from chronic diseases. To our knowledge, no studies have previously found this association among healthy and asymptomatic subjects.

Physical activity is a complex behavioral pattern, and choosing a tool to assess it is challenging. Accordingly, it has yet to be established what would constitute a reasonable gold standard method. Doubly labeled water is considered to be one of the best ways for assessing energy expenditure, but it does not have the capacity to measure the duration, frequency and intensity of activity-related energy expenditure. Accelerometers have been considered to be the tool that has the greatest capability for assessing LPADL. They are precise enough to quantify the physical activity and are cheap enough for use in large epidemiological studies. They have been used as the instrument of choice for validating physical activity questionnaires.^23^ Since there is no defined gold standard method for measuring LPADL, triaxial accelerometry has been recognized as the best method for validating other methods, e.g. the six-minute walk test in the present study.

In our previous study, we found that the six-minute walk test can be described as a moderate to high-intensity exercise in which V'O_2_ and HR of approximately 80% of the maximum may occur. Furthermore, the peak V'O_2_ in CPET was accurately predicted by the six-minute walking distance (R^2 ^= 0.76), through the equation derived.^24^ Although this tool is suitable for evaluating the functional exercise capacity of the majority of middle-aged and older adults, some studies have failed to demonstrate any association between self-reported physical activity and six-minute walking distance.^25^ ^26^ This inconsistency may be due to self-reported physical activity. In the present study, the six-minute walking distance was significantly correlated with LPADL, as evaluated through accelerometry.

The ability to walk as far as possible is associated with better health status among patients with chronic diseases and asymptomatic older adults.^27^ We found that a six-minute walking distance ≤ 511 m was the best predictor of physical inactivity, although this absolute value could be questioned, since it is influenced by factors such as height, weight and age. However, the absolute distance proved valid for predicting LPADL because the six-minute walking distance as a percentage of the predicted value was also reduced in individuals who walked less than 511 m in the six-minute walk test.

Our findings from asymptomatic subjects were similar to those described for patients with chronic obstructive pulmonary disease (COPD). Steele et al.^28^ used a triaxial accelerometer to measure LPADL in 47 patients with COPD. The authors observed a significant correlation between the six-minute walking distance and accelerometry (r = 0.74). Pitta et al.^29^ also used a triaxial motion sensor among 50 patients with COPD, and a strong correlation between walking time in daily life and six-minute walking distance (r = 0.76) was observed. In the same study, patients who walked less than 400 m in the six-minute walk test were considered to be extremely inactive in daily life.

According to univariate analysis in the present study, body fat mass was able to predict physical inactivity. The sensitivity for predicting physical inactivity was 85% among individuals with body fat mass > 25%. However, in the multivariate analysis, body composition was no longer a significant predictor of physical inactivity. In fact, the area under the curve and the specificity of 54% that was found may not be considered to be promising results. This low specificity reflects the inability of body fat mass determinations to identify physical inactivity among individuals with values ≤ 25%. We may suggest that body fat mass is not a good predictor for physical inactivity, since adiposity relates to multiple factors, such as diet, lifestyle, metabolism, genetics and socioeconomic level.^30^

Muscle function was not able to determine physical inactivity in the present study, and our results are in agreement with the previous literature. Garcia et al.^31^ reported that there was only a moderate correlation between these variables. Likewise, V'O_2_ obtained during CPET was not selected as a determinant of physical inactivity in the present study. The walking velocity reached during the six-minute walk test possibly reproduces the LPADL of the general population better, and therefore, the six-minute walking distance is more suitable for predicting physical inactivity than is the peak V'O_2_ obtained at the end of the treadmill CPET.^32^

This study has limitations that need to be considered. The LPADL can be determined through sociocultural and economic factors that were not evaluated in this study and were not adjusted for, in the multiple logistic regression model. Triaxial accelerometry is not the gold standard method for assessing the LPADL, and therefore its use may have introduced bias into our analysis. However, a gold standard method remains to be established.^23^ This instrument is most often used as a reference in validating other methods. For this reason, we suggest caution when extrapolating our results. Nevertheless, we are confident about the usefulness of the six-minute walking distance for screening for physical inactivity in the general population.

Functional exercise capacity (i.e. the six-minute walk test) is a suitable strategy for screening for physical inactivity among adults. The six-minute walk test should be included in epidemiological studies as a simpler and cheaper tool for screening for physical inactivity.
